# The effect of diabetes mellitus on differentiation of mesenchymal stem cells into insulin-producing cells

**DOI:** 10.1186/s40659-024-00502-4

**Published:** 2024-05-02

**Authors:** Omar I. Badr, Mohamed M. Kamal, Shohda A. El-Maraghy, Heba R. Ghaiad

**Affiliations:** 1 Pharmacology and Biochemistry Department, Faculty of Pharmacy, The British University in Egypt, Cairo, Egypt; 2 Biochemistry Department, Faculty of Pharmacy, Cairo University, Cairo, Egypt; 3 Drug Research and Development Group, Health Research Center of Excellence, The British University in Egypt, Cairo, Egypt; 4 Biochemistry Department, Faculty of Pharmacy, Ain Shams University, Cairo, Egypt

**Keywords:** Diabetes mellitus, Mesenchymal stem cells, Differentiation, Insulin-producing cells, Adipose tissue

## Abstract

**Background:**

Diabetes mellitus (DM) is a global epidemic with increasing incidences. DM is a metabolic disease associated with chronic hyperglycemia. Aside from conventional treatments, there is no clinically approved cure for DM up till now. Differentiating mesenchymal stem cells (MSCs) into insulin-producing cells (IPCs) is a promising approach for curing DM. Our study was conducted to investigate the effect of DM on MSCs differentiation into IPCs in vivo and in vitro.

**Methods:**

We isolated adipose-derived mesenchymal stem cells (Ad-MSCs) from the epididymal fat of normal and STZ-induced diabetic Sprague–Dawley male rats. Afterwards, the in vitro differentiation of normal-Ad-MSCs (N-Ad-MSCs) and diabetic-Ad-MSCs (DM-Ad-MSCs) into IPCs was compared morphologically then through determining the gene expression of β-cell markers including neurogenin-3 (Ngn-3), homeobox protein (Nkx6.1), musculoaponeurotic fibrosarcoma oncogene homolog A (MafA), and insulin-1 (Ins-1) and eventually, through performing glucose-stimulated insulin secretion test (GSIS). Finally, the therapeutic potential of N-Ad-MSCs and DM-Ad-MSCs transplantation was compared in vivo in STZ-induced diabetic animals.

**Results:**

Our results showed no significant difference in the characteristics of N-Ad-MSCs and DM-Ad-MSCs. However, we demonstrated a significant difference in their abilities to differentiate into IPCs in vitro morphologically in addition to β-cell markers expression, and functional assessment via GSIS test. Furthermore, the abilities of both Ad-MSCs to control hyperglycemia in diabetic rats in vivo was assessed through measuring fasting blood glucose (FBGs), body weight (BW), histopathological examination of both pancreas and liver and immunoexpression of insulin in pancreata of study groups.

**Conclusion:**

Our findings reveal the effectiveness of N-Ad-MSCs in differentiating into IPCs in vitro and controlling the hyperglycemia of STZ-induced diabetic rats in vivo compared to DM-Ad-MSCs.

**Graphical Abstract:**

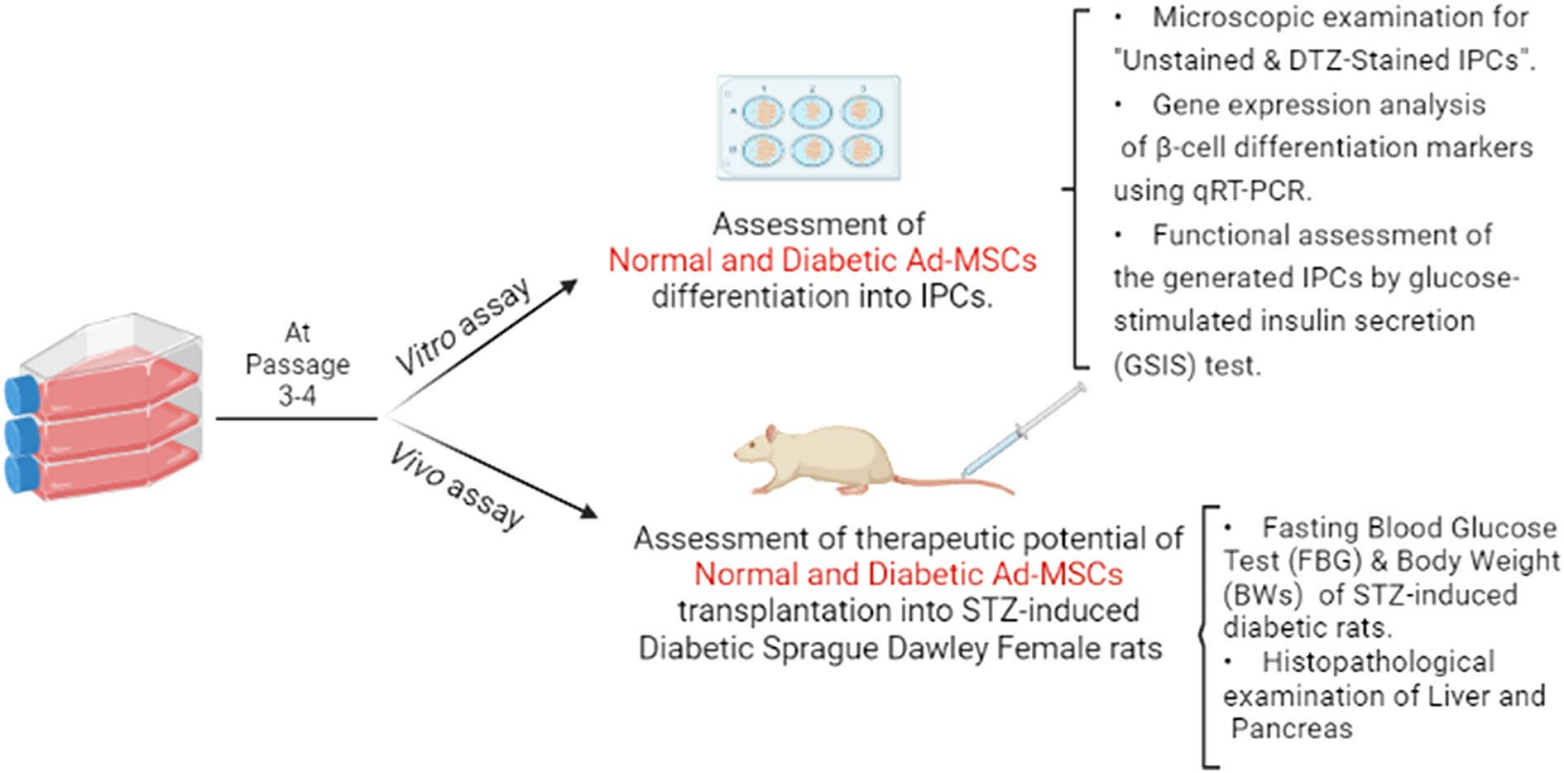

**Supplementary Information:**

The online version contains supplementary material available at 10.1186/s40659-024-00502-4.

## Introduction

Diabetes mellitus (DM), one of the world’s most serious epidemics. DM is a syndrome that is recognized as a class of disease associated with chronic signs and symptoms of hyperglycemia [1]***.*** Furthermore, DM affects various organ systems and provokes a variety of vascular and nonvascular complications, which are the leading cause of premature death [2, 3]*.* Type 1 diabetes mellitus (T1DM), type 2 diabetes mellitus (T2DM), and gestational diabetes mellitus are among the most common types, however, other types are considerably less common [1]*.*

Many immune cells are involved in the multifactorial and polygenic pathogenesis of insulin-dependent DM, commonly known as T1DM. During the clinically silent insulitis, mononuclear cells infiltrate the pancreatic islets of Langerhans, and subsequently, T lymphocytes destroy insulin-producing β cells. Natural killer (NK) cells also contribute to T1DM as both causative and protective cells, but the molecular mechanism by which they contribute to T1DM is still unknown [4]*.* While T2DM, which represents 85–95% of all cases, is associated with insulin resistance (IR) within insulin-responsive tissues as well as a decline in insulin production from pancreatic β cells [3, 5]*.*

Insulin injections or wearing advanced patches that can provide insulin at an automated programmed time are common treatments for T1DM. However, such delivery systems do not eliminate hyperglycemic complications such as nephropathy and ketoacidosis. Additionally, the dosage and onset of action of administered insulin are completely different from the physiological level of insulin action [6, 7]. Treating T1DM by pancreatic transplantation is also limited due to the lack and shortage of tissue donors in addition to the risk of immunological rejection [6, 8, 9]. As a result, studies are being conducted to see if the differentiation potential of mesenchymal stem cells (MSCs) may be used to treat T1DM [10–13].

MSCs are stromal cells with the ability to self-renew and differentiate into many lineages. They can be extracted from various tissues, including the umbilical cord, bone marrow, and adipose tissue. This is owing to the ease with which they may be harvested and the quantity obtained, making these sources the most feasible for experimental and therapeutic purposes [14–16]. Furthermore, the latest studies have revealed that MSCs implantation could be used as a recent therapeutic intervention for DM by lowering blood glucose levels through paracrine impacts rather than direct trans-differentiation into insulin-producing cells (IPCs) [17–20]. Nowadays, using IPCs is considered a new approach to an effective therapy for both kinds of DM which is mainly derived from the multipotent character of MSCs [6]. Cell-based therapies also demonstrated a potential approach to T2DM treatment through generating an infinite supply of insulin-producing cells, mending β cell function, modifying metabolism, and alleviating immunological dysfunction [21, 22]. MSCs have been considered a favorite type of stem cell among researchers because of their hypo-immunogenic properties, multipotent character, and relative abundance in human tissue [6]. MSCs can develop into IPCs in T2DM. Key transcription factors such as neurogenic differentiation 1 (NEUROD1), NK2 related transcription factor related, locus 2 (NKX2-2) were activated to govern the differentiation program [23–25]*.* Some transcription factors, such as Isl-1 and Pax-6, were found to be expressed in adipose derived mesenchymal stem cells (Ad-MSCs), indicating that Ad-MSCs can differentiate into IPCs to cure diabetes [26]*.*

Because of the effects of DM on Ad-MSCs, studies have shown that T2DM can limit the feasibility of autologous stem cell therapy. In addition, DM showed alteration in gene expression in Ad-MSCs in a T2DM rat model, including MSC-specific markers, stem markers, stem cell maintenance molecules, and cellular mobility molecules [3, 27]. The diabetic condition has an impact role on the reactive oxygen species (ROS) system. ROS is implicated in the modulation of cell physiological differentiation, proliferation, and migration, as well as illnesses such as DM, hypertension, and several degenerative diseases [28, 29]. The metabolic phenotype of DM altered the immunomodulatory properties of Ad-MSCs. Ad-MSCs derived from obese and T2DM compared to lean-derived Ad-MSCs displayed an increase in expression and secretion of inflammatory cytokines such as interleukin (IL)-1β, IL-6, tumor necrosis factor-alpha (TNF-α) [30]***.***

In this study, we aimed to investigate the difference in the characteristics of the Ad-MSCs isolated from normal non-diabetic rats in comparison to diabetic ones. In addition, we assessed the abilities of normal adipose MSCs (N-Ad-MSCs) and diabetic adipose MSCs (DM-Ad-MSCs) to differentiate into IPCs in vitro and their abilities to control hyperglycemia in diabetic rats in vivo.

## Material and methods

The experimental animal protocol for this study was designed and reported in accordance with the Animal Research: Reporting of In Vivo Experiments (ARRIVE) guidelines, and in compliance with the regulation and guidelines of the Ethical Committee of the Faculty of Pharmacy, The British University in Egypt which approved the study protocol BC (EX-2103) on March 17th, 2021. Additionally, all the procedures were reviewed and approved by research ethics committee of Faculty of Pharmacy, Cairo University (REC-FOPCU), Cairo, Egypt, approval number: BC (B2914) on January 25th, 2021. The current work was approved under the name “The effect of diabetes mellitus on mesenchymal stem cells and their potential to differentiate into insulin-producing cells”.

### Induction of type 1 DM by STZ injection

20 Sprague–Dawley male healthy rats, 12 weeks of age, weighing 200–300 g were used (Rats with HbA_1C_ test over 5.7% or with a pervious access for high-fat foods were excluded). The sample size was determined using G*power software to achieve minimum power of 80%. The animals were kept at 23 °C under a 12 h light–dark cycle, with free access to food and water. Rats fasted for 6–7 h from food, but the water was freely allowed then the fasting blood glucose (FBG) test was measured. The test was performed using chemically treated, disposable glucose oxidase reagent strips (Bionime GS100), then rats with FBG 110 mg/dl (6.1 mmol/L) or below were selected to undergo the experiment. DM in 12 rats was induced by intraperitoneal injection of streptozotocin (STZ) (50 mg/kg) in citrate buffer as described previously [31]. STZ (Alfa-Aeser) was dissolved in freshly prepared 0.1 M citrate buffer. DM was verified 72 h after STZ injection. Rats with FBG over 200 mg/dL (11.1 mmol/L) were considered diabetic rats and were used in the subsequent experiment. On the other hand, 6 fasted rats with FBG 85–110 mg/dl were considered control (normal non-diabetic rats).

### Isolation of Ad-MSCs from the epididymis of normal non-diabetic (N-Ad-MSCs) and diabetic rat (DM-Ad-MSCs)

The isolation protocol was performed as described previously with modifications [32, 33]. 6 Sprague–Dawley male rats were selected from the diabetic rats’ group after 14 days of diabetes initiation versus 6 normal non-diabetic rats. The rats were anesthetized using intraperitoneal injection of ketamine hydrochloride (33 mg/kg body weight) and xylazine hydrochloride (13 mg/kg body weight) as explained before [34, 35] and then euthanized by cervical dislocation. The anesthetized rats were placed and used to incise the abdomen area along the peritoneal cavity to get epididymal tissue located over testes of the rat. In an aseptic area; the collected epididymal tissues were cut into small pieces and washed 4–5 times with phosphate buffered saline (PBS) then mixed with an equal volume of 0.1% collagenase solution then placed within a water bath (80 rpm/37 °C/30–45 min) followed by vortexing and centrifuging 2 times at 1200 rpm (300 ×*g*) for 5 min., The lower layer of stromal vascular fraction (SVF) was re-suspended in 1% bovine serum albumin (BSA) and centrifuged at 1200 rpm (300 ×*g*) for 5 min. The pellet was re-suspended with Dulbecco's modified eagle medium/nutrient mixture F-12 (DMEM/F12) media (Lonza, Switzerland) supplemented with 10% fetal bovine serum (FBS) (Gibco, Thermo Fisher), 1X antibiotic/antifungal (100 U/mL penicillin, 0.25 µg/mL amphotericin B, and 100 µg/mL streptomycin) (Gibco, Thermo Fisher), and 2 mM L-glutamine (Gibco, Thermo Fisher). Isolated cells within a 25 cm^2^ flask named (P0), normal-Ad–MSCs (N-Ad-MSCs) that derived from normal rat or (P0), diabetic-Ad-MSCs (DM-Ad-MSCs) that derived from STZ-induced diabetic rat and incubated at 37 °C /5% CO_2_. Cells with 80- 90% confluency have undergone passaging using 0.25% trypsin-ethylenediaminetetraacetic acid (EDTA) (Gibco, Thermo Fisher).

### Characterization of Ad-MSCs

One of the characteristic criteria of MSCs is their ability to attach to plastic surfaces with flat fibroblast-like shaped cells (MSCs-like cells) and also their proliferation and expansion under specific cultural conditions in addition to their multilineage differentiation abilities [36]*.*

#### Flow cytometry immuno-phenotyping assays

N-Ad-MSCs and DM-Ad-MSCs were collected as cell pellets at P3 and then washed with PBS twice. The recommended amounts of monoclonal antibodies labeled with fluorescein-isothiocyanate (FITC) for mesenchymal markers CD90 or CD105 or phycoerythrin (PE) labeled for CD34 (Stem cells Technologies, USA) as hematopoietic marker, were used, while unstained cells were used as controls. The antibodies, either alone or in combination of 2 different labelling, were mixed with 1 × 10^5^ cells and suspended in 500 μLs of fluorescence-activated cell sorting (FACS) buffer and analyzed by a CYTOMICS FC 500 Flow cytometer (Beckman Coulter, USA).

#### Multilineage differentiation assays

The ability of cells to differentiate into adipocytes, osteocytes, and chondrocytes is one of the International Society for Cell and gene therapy (ISCT)'s criteria for defining MSC [36]. The mesenchymal stem cell functional identification kit (R&D Systems Inc., USA) was used to induce the multilineage differentiation of the MSCs then the stained cells were observed under the microscope (Olympus inverted microscope, Japan) and the kit was used as follows.

##### Adipogenic differentiation assay

Cells at P3 were seeded in a 24-well tissue culture plate with a density of 3.7 × 10^4^ cells in each well and supplemented with complete DMEM/F12 Media, 10% FBS, and 1X antibiotic/antifungal. The stromal media was removed from each well every 2–3 days till it reached 100% confluency and adipogenic induction media was added in HG-DMEM (Lonza, Switzerland) for 21 days while there were Ad-MSCs cultured in growth complete DMEM/F12 medium deprived of differentiation factors serve as a control. After this specified period, the cells were washed with PBS and fixed by incubation in 10% buffered formalin for 10 min at room temperature (RT). The cell monolayer was stained with a working solution of 0.5% Oil Red staining (Sigma-Aldrich) which appeared as lipid vacuoles under a microscope.

##### Osteogenic and chondrogenic differentiation assay

Cells at P3 with a density of 7.4 × 10^3^ cells/well were cultured in growth complete DMEM/F12 medium and incubated for 3 days until they reached 70–80% confluency. The cells were grown in either osteogenic or chondrogenic induction media respectively for 21 days while the other non-differentiated MSCs cultured in growth complete DMEM/F12 media that were deprived of differentiation factors to act as a control. After the differentiation period, the cell monolayer was washed with PBS and fixed by incubation in 4% paraformaldehyde (PFA) for 20 min at RT then covered with 2% Alizarin Red (Sigma-Aldrich) for staining osteocytes that appeared as calcium-rich extracellular matrix while 0.1% Alcian blue 8GX stain (Sigma-Aldrich) in 3% glacial acetic acid was used for staining chondrocytes, especially sulfated proteoglycan.

### In vitro differentiation of Ad-MSCs into insulin-producing cells (IPCs)

A simple differentiation protocol was carried out as described previously with modifications [33, 37]**.** A schematic presentation of this protocol is illustrated in Fig. 2A. Briefly**,** N-Ad-MSCs and DM-Ad-MSCs at passages 3–4 were seeded in 6-well plates and 12 well plates with equal density of 1 × 10^6^ cell/plate and supplemented with a complete DMEM/F12 medium and left for 3 days to reach 80–90% confluency. IPCs differentiation cells were passed by three phases as shown in Fig. 3 while other cells were deprived of differentiation factors to act as a control Ad-MSCs. Phase I: cells were grown for 2 days by pre-induction media; DMEM/F12 supplemented with 10% FBS, 1X antibiotic/antifungal, 2 mM L-glutamine, 10 mM nicotinamide (NA) and 1 mM β-mercaptoethanol (β-ME). Phase II: Day 3 of differentiation; media of cells was replaced by another day of HG-DMEM supplemented with 2.5% FBS, 1X antibiotic/antifungal, 2 mM L-glutamine, 10 mM (NA) and 1 mM (β-ME). Two cell pellets were collected at this stage and named (D3 IPCs). Phase III: cells were incubated with the final differentiation induction media consisting of HG-DMEM supplemented with 2.5% FBS, 1% Antibiotic/antifungal, 2 mM L-glutamine, 10 mM NA, 1 mM β-mercaptoethanol and 10 nM exendin-4 (Sigma-Aldrich) for 7 days. At the end of the specified period, cell pellets were collected and named (Final IPCs), and cells were assessed by dithizone (DTZ) staining, β cell marker expression analysis, and glucose-stimulated insulin secretion **(**GSIS) as will follow in this section.

### Assessment of differentiation of N-Ad-MSCs and DM-Ad-MSCs into IPCs

#### Dithizone (DTZ) staining

DTZ staining was performed to confirm IPCs differentiation. DTZ is a zinc chelating agent that identifies β cells containing zinc. The pancreatic islets which are positive with this staining (red color stained with crimson red in the solution) account for the accomplishment of differentiation into β cells or insulin-producing cells [38]. A stock solution was prepared by dissolving 50 mg DTZ in 5 mL dimethyl sulfoxide (DMSO) and stored at − 20 °C in a dark area then mixed and diluted with a complete culture medium; HG-DMEM supplemented with 5% FBS, 1X antibiotic/antifungal and 2 mM L-glutamine then the working solution was filter sterilized. The culture dishes were incubated with DTZ solution for 2h/37 °C, after discarding their media and then washed subsequently three times with PBS while undifferentiated Ad-MSCs were used as a negative control. Differentiated IPCs versus undifferentiated were observed with an inverted microscope (Olympus, Japan) equipped with a digital camera.

#### Gene expression assay using qRT-PCR

##### RNA extraction

Cell pellets (from normal undifferentiated Ad-MSCs, D3 differentiation, or final differentiated IPCs cells) were collected after trypsinization and stored at – 80 °C till further use. The pellets were suspended in 1 mL TRIzol^®^ reagent (Invitrogen). The lysed samples were incubated for 5 min then 200 µLs of chloroform were added. Following centrifugation, the mixture was separated into three layers while RNA remains exclusively in the upper aqueous phase. 500 µLs of 100% isopropanol were added to the collected aqueous phase then incubated at R.T and after centrifugation of 12,000*×g* for 30 min at 4 °C, a gel‐like pellet of RNA precipitate was washed with 1 mL 80% ethanol followed by centrifugation 5 min at 9600*×g* at 4 °C. The RNA pellets were left for air drying. After drying, RNA pellets were dissolved in RNase-free water and quantified by determining the optical densities (OD) at 260 nm and 280 nm using nuclease-free water as blank.

##### cDNA synthesis, qRT-PCR using SYBR Green Master Mix

cDNA synthesis was done according to the manufacturer’s instructions of the Verso ™ kit (Thermo Scientific, USA). 0.5 µg of total RNA was used for cDNA synthesis. The master mix with RNA was passed through a cycling program of 50 °C for 30 min for one cycle, followed by an inactivation cycle of 95 °C for 2 min for one cycle using sensoquest labcycler thermal cycler, USA. The cDNA was diluted by nuclease-free water to a final concentration of 2 ng/µl and stored at − 20 °C for qRT-PCR.

##### Quantitative reverse transcriptase polymerase chain reaction (qRT-PCR)

The qRT-PCR reaction was done for each target gene using a Maxima Sybr green Master Mix (Thermo Fisher, USA) according to the manufacturer’s instructions. All measures were done in triplicates. The sets of primers used were shown in Table 1**.** Neurogenin-3 (Ngn-3), homeobox protein Nkx6.1 (Nkx6.1), musculoaponeurotic fibrosarcoma oncogene homolog A (MafA), and insulin-1 (Ins-1) were measured**.** Those β-cell differentiation markers are considered as critical transcription factors for β-cell differentiation and maturation [39–41]. All qRT-PCR reactions were done in the Step One Plus RT-PCR system (Applied Biosystems, USA) using the default program and dissociation curve. Relative RNA expression was done using the 2^−ΔΔCt^ method using β-actin as a housekeeping gene.

**Table 1:** Sequence of primers used for the RT-qPCR

Reverse primer 5′–3′	Forward primer 3′–5′	Genes
GAGCTTCCTCGATGTCCCTC	ACCATCCAAGTGTCCCAAGA	Ngn-3
CTCCAGTGCCAAGGTCTGA	CACCTTTGTGGTCCTCACCT	Ins-1
CCGCCAACTTCTCGTATTTC	TTCAGCAAGGAGGAGGTCAT	Mafa
ATCTCGGCTGCGTGCTTCTT	ACACCAGACCCACATTCTCCG	NKX6.1
AACACAGCCTGGATGGCTAC	TGGAGAAGATTTGGCACCAC	β-actin

##### Functional assessment of the generated IPCs by glucose-stimulated insulin secretion (GSIS) test

Two glucose concentrations (low glucose (LG) = 2 mM, high glucose (HG) = 20 mM) in Kreb’s Ringer bicarbonate (KRB) buffer solution was used to determine in vitro potency of the IPCs and whether the insulin release of the generated IPCs was glucose-dependent as previously described with modifications [42]. A sterile filtered LG and HG-KRB buffer preparations were prepared as follows: 18 mg glucose for 50 mL KRB to prepare LG-KRB buffer mixture while 180 mg glucose for 50 mL KRB to prepare a HG-KRB buffer mixture. After adjusting the pH of the buffer using 1N HCl to be (7.25–7.35), 0.1% BSA was freshly added. The supernatants from the medium of the induced IPCs for LG and HG concentrations were then harvested for quantification of the released insulin using an enzyme-linked immunosorbent assay (ELISA) as previously described by [43].

Following the induction protocol, the IPCs were gently washed twice with PBS and once with LG-KRB. The IPCs were then cultured for 1 h with 300 µLs LG-KRB/well. The IPCs were incubated with either LG or HG-KRB mixture for an additional 1 h and the supernatant was collected and kept at – 80 °C to be examined and the secreted insulin by IPCs was analyzed using an ELISA kit (Cloud Clone, USA) according to the manufacturer's instructions.

### In vivo assessment of the therapeutic potential of N-Ad-MSCs and DM-Ad-MSCs in STZ-induced diabetic rats

Induction of DM by STZ injection in rats

T1DM in 24 female Sprague–Dawley healthy rats was induced by subcutaneously injecting STZ (50 mg/kg in Citrate buffer). The same inclusion and exclusion criteria described earlier in this study were applied. On the other side, 6 normal non-diabetic rats were selected to be as a control group. FBG and body weight were measured after 72 h of STZ induction. 10 days after induction of diabetes; 18 STZ rats were randomized using simple randomization method into the 3 groups; and then they were categorized into three groups; STZ + PBS group (6 non-treated STZ-induced diabetic rats), STZ + DM-Ad-MSCs group (6 STZ-induced diabetic rats treated with DM-Ad-MSCs in PBS), STZ + Normal-Ad-MSCs group: (6 STZ-induced diabetic rats treated with N-Ad-MSCs in PBS).The animals groups were blindly checked for FBG and BWs by an independent technician.

Transplantation of Ad-MSCs

On the day of injection, each fasted rat from STZ + DM-MSCs and STZ + Normal–MSCs group received 1–1.5 × 10^6^ freshly trypsinized P3-P4 (DM-Ad-MSCs) and (N-Ad-MSCs), respectively, where cells were suspended in 1 mL PBS. Cells suspended in PBS were transplanted into tail veins using a 1 mL 100-unit 27-gauge insulin syringe. FBG and BW were measured every 7 days after the day of transplantation for 35 days only. Transplantation was done by the same experimenter for all animals and blindly regarding the groups.

### Histopathological examination of some organs of the treated diabetic rats

To investigate the in vivo effects of both N-Ad-MSCs and DM-Ad-MSCs treatments in STZ-induced diabetic rats, autopsy samples were collected on day 35 of transplantation from different affected organs such as livers and pancreas of rats in different groups, as well as a control group to be examined by histopathology. Isolated tissue sections were immediately preserved in 10% buffered formalin for 24–48 h and stored at room temperature. The tissues were then sectioned, fixed on microscope slides, and embedded with paraffin. The sections were stained using hematoxylin and eosin (H&E) stain for blinded randomized examination by two independent researchers. The microscopic examination of stained sections was performed by LABOMED Fluorescence microscope LX400, and LABOMED camera software (Labomed, USA*).* The images were taken with magnifications 10× and 40× for clarification. All histopathological examinations were done blindly regarding the study groups.

### Immunostaining of insulin in pancreata of the treated diabetic rats

Paraffin-embedded tissues were deparaffinized and rehydrated through graded alcohol series. Afterwards, peroxidase blocking serum was added for 10 min, then washed with PBS. This was followed by incubation with protein blocking serum for 10 min. Staining was done by adding anti-insulin antibody (Diagnostic Biosystems, USA) and incubating in moist chamber for 30 min at room temperature, followed by adding the pre-diluted biotinylated secondary antibody to each section for 45 min, then washed with PBS. Then horse radish peroxidase conjugated streptavidin was added for 20 min followed by washing with PBS. Finally, substrate/ 3,3′-diaminobenzidine (DAB) mixture, prepared immediately before use, was added and incubated for 5–10 min followed by rinsing with distilled water. Sections were then counterstained using Harris’s hematoxylin. The microscopic examination was performed by LABOMED Fluorescence microscope LX400, and cells were counted in 3 high power fields (HPF) per group.

### Statistical analysis

All data are expressed as mean ± standard error of the mean (SE). The comparisons between β cell markers expression and the quantification of immunostaining of insulin were done by using a one-way analysis of variance (ANOVA) and the Tukey post hoc test. Comparison of GSIS was done by t-test while the comparisons between groups in the in vivo study was done by two-way ANOVA. All means were considered significantly different with *p-value* < 0.05. All figures and statistical analyses were done using Graph Pad Prism (Version 6, USA).

## Results

### Isolation, culture, characterization of the cells isolated from both N-Ad-MSCs and DM-Ad-MSCs

**Fig. 1:**
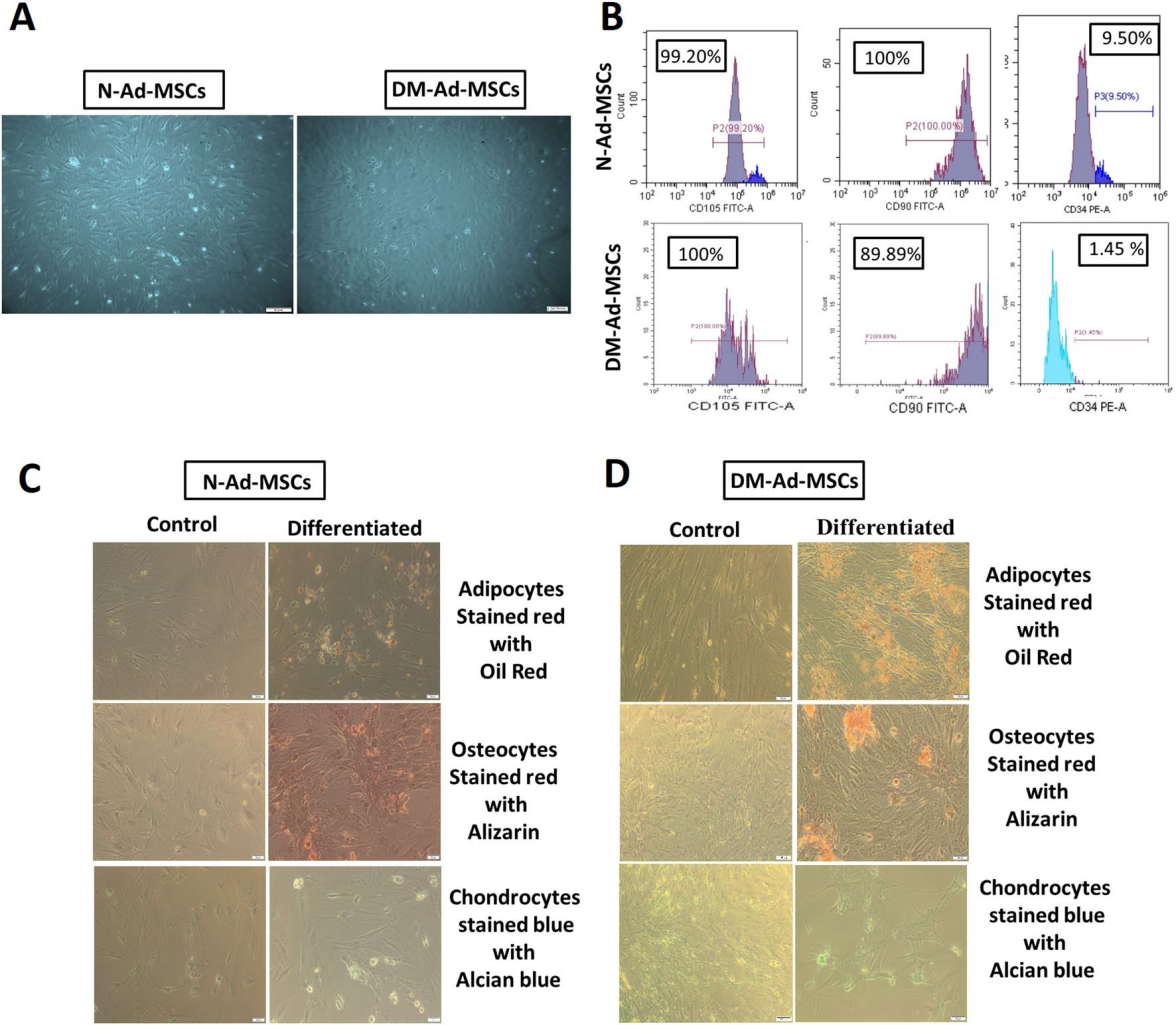
**A** Phase contrast images of cultured Ad-MSCs; either freshly isolated N- Ad-MSCs (Day7, P0) showing a homogenous population of fibroblast-like shaped cells (left), or freshly isolated DM-Ad-MSCs (Day 7, P0) showing a homogenous population (right). **B** Immunophenotyping of N-Ad-MSCs (upper) showing positive expression of mesenchymal markers (CD105-FITC: 99.20%, CD90-FITC: 100%) and negative expression of hematopoietic markers (CD34-PE: 9.50%, the blue peak represents positive PE-stained cell). While the immunophenotyping of DM-Ad-MSCs (lower panel) showed positive expression of mesenchymal markers (CD105-FITC 100%, CD90-FITC 89.89%) and almost negative expression of hematopoietic markers (CD34-PE 1.45%). **C** and **D** Multilineage differentiation of N- and DM-Ad-MSCs: **C** N-Ad-MSCs differentiated into adipocytes; showing oil red staining, osteocytes; showing alizarin red staining and chondrocytes; showing alcain blue staining (left) as compared to control cells (right). **D** DM-Ad-MSCs differentiated into adipocytes, osteocytes, and chondrocytes (right) as compared to control cells (left). *N-Ad-MSCs* normal adipose MSCs, *DM-Ad-MSCs* diabetic adipose MSCs, *FITC* Fluorescein isothiocyanate, *PE* phycoerythrin

Isolated N-Ad-MSCs and DM-Ad-MSCs grew as plastic adherent flat fibroblast-like shaped cells (MSCs-like cells) forming a homogenous cell population in culture as shown in Fig. 1A. Therefore, both isolated cells fulfilled the first criterion of MSCs [36]. Both DM- and N- Ad-MSCs were able to proliferate and expand regularly. This indicates that Ad-MSCs can be readily isolated in abundant amounts from rat epididymal fats.

### Immunophenotyping

The second criterion of the MSCs is done by immunophenotyping of the CDs that were expressed on these cells using flow cytometry. Immunophenotyping assessment revealed that the majority of isolated N-Ad-MSCs expressed MSCs markers, where 99.2% of isolated N-Ad-MSCs expressed CD105, while 100% of cells expressed CD90. In addition, these cells were almost negative for hematopoietic markers (CD34 positive cells = 9.50%) as shown in Fig. 1B, upper panel. On the other hand, immunophenotyping of the isolated DM-Ad-MSCs showed a positive expression for MSCs characteristic CDs including CD105 and CD90 (99.9% and 89.89% positive cells respectively). In addition, these cells were almost negative for hematopoietic CDs such as (CD34 positive cells = 1.45%) Fig. 1B, lower panel. These results indicate that either N-Ad-MSCs or DM-Ad-MSCs showed similar homogenous mesenchymal phenotypic population.

### Multilineage differentiation (adipogenic, osteogenic, and chondrogenic differentiation) of normal and diabetic Ad-MSCs

As a functional assay to confirm the isolated cells' MSC identity, we investigated the multilineage differentiation potential of both N-Ad-MSCs and DM-Ad-MSCs.

As shown in Fig. 1C and D, both N- and DM-Ad-MSCs could differentiate into adipocytes, osteocytes, and chondrocytes while control undifferentiated cells preserved their fibroblast-like form and failed to be stained with any differentiation stains. The adipogenic cells with rounded lipid vacuoles stained with oil red stain, in addition to the osteogenic cells that revealed a calcium-rich extracellular matrix stained with alizarin red while the chondrogenic cells had a cuboidal morphology with sulfated proteoglycan which was stained with alcian 8GX blue.

Accordingly, it can be concluded that mesenchymal lineage differentiation potential of N-Ad-MSCs and DM-Ad-MSCs were the same. In fact, both the isolated N-Ad-MSCs and DM-Ad-MSCs displayed a homogenous stem cell population that exhibits all MSC characteristics.

### Assessment of the in vitro differentiation of N-Ad-MSCs and DM- Ad-MSCs into IPCs

**Fig. 2:**
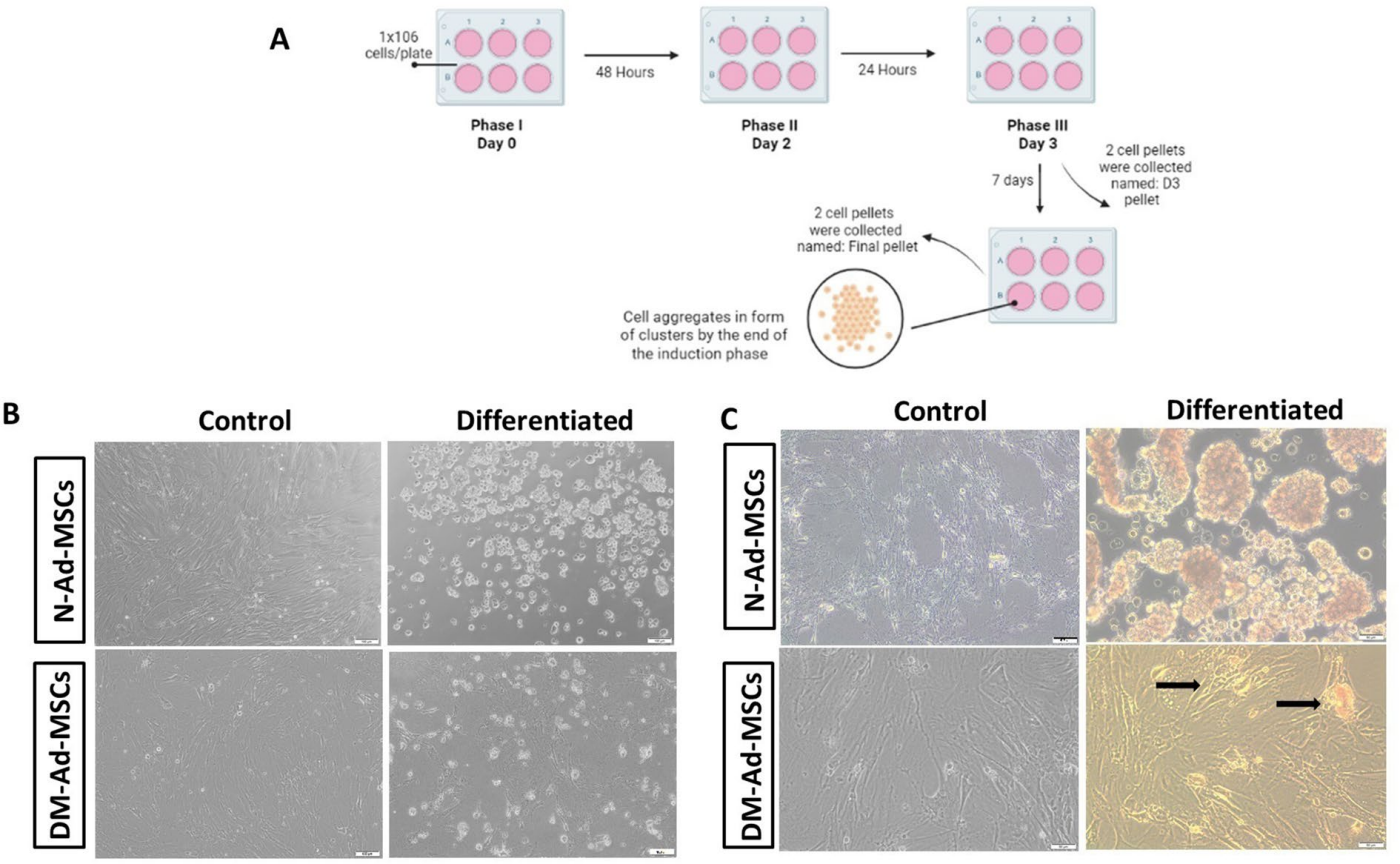
Morphological assessment of IPCs generated from N-Ad-MSCs and DM-Ad-MSCs. **A** Schematic presentation of the differentiation protocol (Phase I, II, III). **B** Phase contrast images of unstained differentiated N-MSCs and DM-MSCs into IPCs; upon differentiation, both N-Ad-MSCs (upper) and DM-Ad-MSCs (lower panel) aggregate to form clusters in contrast to control N-Ad-MSCs and DM-Ad-MSCs which retain fibroblast-like morphology (at magnification 10×). **C** Phase contrast images of DTZ-stained differentiated IPCs of N-Ad-MSCs and DM-Ad-MSCs; DTZ-stained IPCs of N-Ad-MSCs (upper) showing crimson red large clusters with different masses as compared to stained IPCs of DM-Ad-MSCs (lower). Both control N-Ad-MSCs and DM-Ad-MSCs retained the unstained fibroblast-like morphology (at magnification 20x)

The potential of N-Ad-MSCs and DM-Ad-MSCs to differentiate into IPCs through the three-step differentiation protocol, as described previously with modifications [33, 37], has been assessed. Concerning the morphological changes, both cell types, to a different extent, gradually lose their fibroblast-like shape following exposure to differentiation protocols, and then they tend to aggregate to form clusters by the end of phase II till the last phase. Moreover, cells begin to detach and grow in suspension in the culture medium. On the other hand, control cells retained their MSC-like morphology throughout the differentiation process. Interestingly, the generated IPCs from differentiated N-Ad-MSCs formed more cells aggregates as compared to those generated from DM-Ad-MSCs indicating more potential of N-Ad-MSCs to generate IPCs Fig. 2B**.**

DTZ staining was applied for the generated IPCs from both N-Ad-MSCs and DM-Ad-MSCs to examine the mass of crimson red-stained cells. As shown in Fig. 2C**,** IPCs were distinctly stained with crimson red by DTZ. The IPCs generated from N-Ad-MSCs showed larger stained cells aggregates as compared to those generated by DM-Ad-MSCs. Meanwhile, the control undifferentiated cells of both cells retained their unstained MSC-like morphology.

#### Gene expression analysis by qRT-PCR

**Fig. 3:**
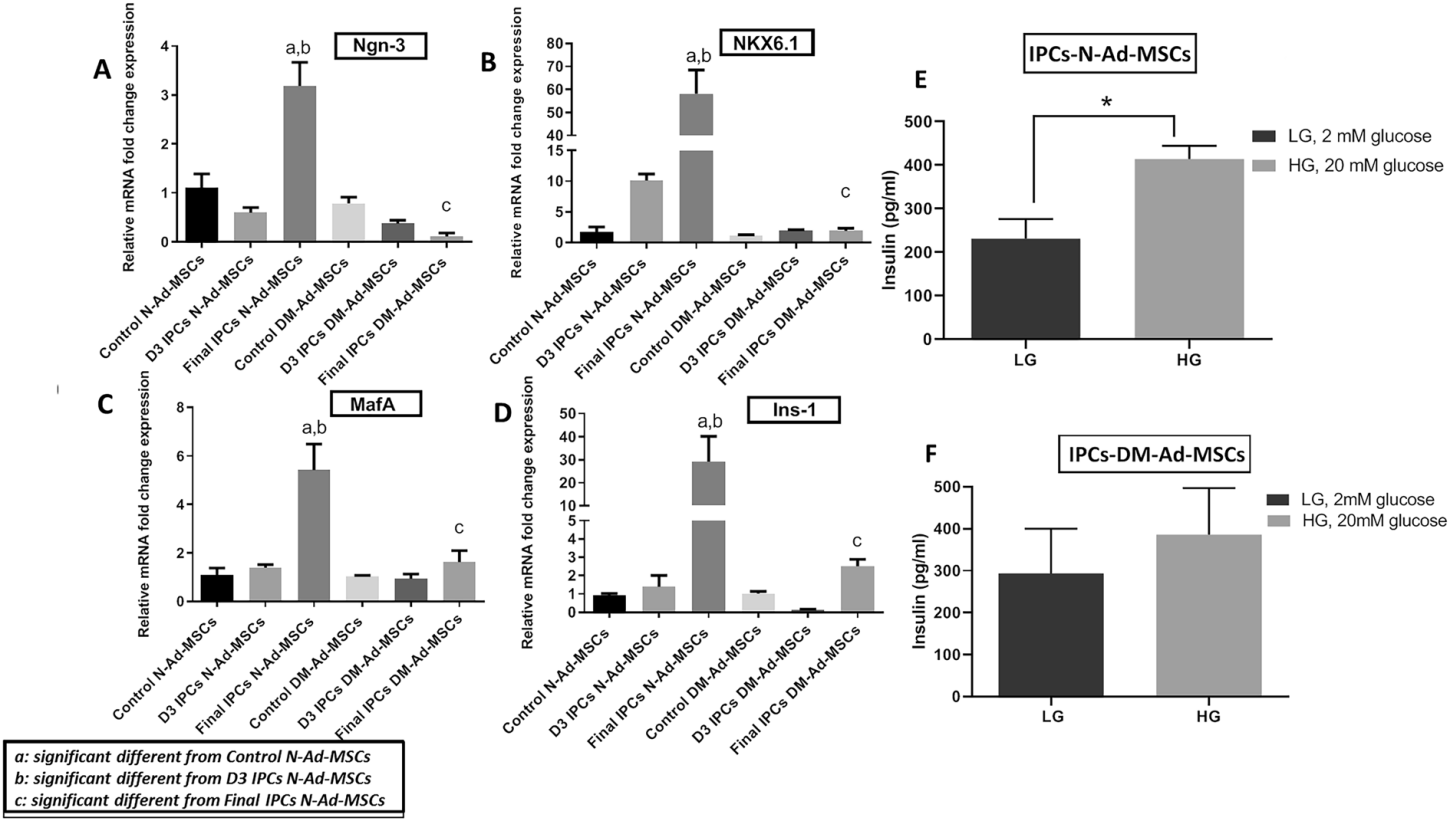
Gene expression of β-cell markers and GSIS upon differentiation of N-Ad-MSC or DM-Ad-MSCs into IPCs: Relative mRNA fold change expression of **A** Ngn-3, **B** NKX6.1, **C** MafA and **D** Ins-1, throughout differentiation protocol of N-Ad-MSCs and DM-Ad-MSCs into IPCs. Insulin release of differentiated IPCs derived from **E** N-Ad-MSCs and **F** in response to LG (2mM) and HG (25mM). **a** significant different from Control N-Ad-MSCs at *p-value* < 0.05. **b** significant different from D3 IPCs N-Ad-MSCs at *p-value* < 0.05. **c** significant different from Final IPCs N-Ad-MSCs at *p-value* < 0.05. * means the significant difference from LG (control) at *p-value* < 0.05. *N-Ad-MSCs* Normal Adipose-MSCs, *DM-Ad-MSCs* Diabetic Adipose-MSCs, *control* Undifferentiated cells, *D3* Day 3 differentiation, *Final* Final generated IPCs

As shown in Fig. 3, all the transcript levels of β-cell differentiation markers in Final IPCs N-Ad-MSCs showed increased expression levels as compared to control cells, an effect failed and impaired to be shown by the IPCs generated by the DM-Ad-MSCs. IPCs N-Ad-MSCs showed higher mRNA relative expression levels of all 4 β-cell pancreatic genes than the differentiated IPCs DM-Ad-MSCs. Figure 3A showed that Ngn-3 showed increased expression levels reaching up to 3 folds in IPCs generated from N-Ad-MSCs while failed to raise in IPCs generated from DM-Ad-MSCs (Control N-Ad-MSCs: 1.10 ± 0.39; D3 IPCs N-Ad-MSCs:0.59 ± 0.14; Final IPCs N-Ad-MSCs: 3.18 ± 0.56, Control: DM-Ad-MSCs: 0.78 ± 0.21; D3 IPCs DM-Ad-MSCs: 0.37 ± 0.07; Final IPCs DM-Ad-MSCs: 0.11 ± 0.07 *p-value* < 0.05).The same pattern was shown by NKX6.1 (Control N-Ad-MSCs: 1.65 ± 0.86; D3 IPCs N-Ad-MSCs: 10 ± 1.46; Final IPCs N-Ad-MSCs: 58.10 ± 14.62; Control DM-Ad-MSCs: 1.05 ± 0.21; D3 IPCs DM-Ad-MSCs: 1.96 ± 0.92; Final IPCs DM-Ad-MSCs: 1.9 ± 0.42 *p-value* < 0.05), Fig. 3B. In addition, MafA was also significantly elevated in final IPCs generated by the N-Ad-MSCs reaching about 6 folds compared to only 2 folds elevation in DM-Ad-MSCs (Control N-Ad-MSCs: 0.77 ± 0.07; D3 IPCs N-Ad-MSCs: 1.39 ± 0.04; Final IPCs N-Ad-MSCs: 5.42 ± 1.31; Control DM-Ad-MSCs: 1.06 ± 0.06; D3 IPCs DM-Ad-MSCs: 0.93 ± 0.05; Final IPCs DM-Ad-MSCs: 1.79 ± 1.1 *p-value* < 0.05)., Fig. 3C. As shown in Fig. 3D, IPCs generated from both N-Ad-MSCs and DM-Ad-MSCs showed an elevation in the expression of Ins-1 with an almost 30-fold increase in N-Ad-MSCs versus only threefold increase in DM-Ad-MSCs as compared to their corresponding controls (Control N-Ad-MSCs: 0.9 ± 0.11; D3 IPCs N-Ad-MSCs: 1.38 ± 0.62; Final IPCs N-Ad-MSCs: 29.2 ± 15.6; DM-Ad-MSCs: Control: 1 ± 0.13; D3 IPCs DM-Ad-MSCs: 0.09 ± 0.04; Final IPCs DM-Ad-MSCs: 2.5 ± 0.38 *p-value* < 0.05). These findings indicate that both cells could differentiate down the pancreatic lineage, to a different extent as N-Ad-MSCs showed higher potentiality towards differentiation into IPCs.

#### GSIS for N-Ad-MSCs and DM-Ad-MSCs derived IPCs

Pancreatic β‐cells produce insulin at a given time when extracellular glucose levels are detected [44]. Therefore, the secretion of insulin in response to glucose is an important feature of β–cells [43]. As shown in Fig. 3E, the IPCs generated by N-Ad-MSCs showed significantly higher insulin secretion upon challenge with higher glucose concentrations (Insulin LG (2 mM): 231.4 ± 45.23 pg/mL; HG (20 mM): 413.8 ± 30.75 pg/mL p-value < 0.05) that showed the sensitivity of IPCs N-Ad-MSCs toward the two different glucose concentration. On the other hand, although IPCs of DM-Ad-MSCs Fig. 3F revealed a slight elevation in insulin response, they did not approach significant levels within low versus high glucose concentration (Insulin LG (2mM): 293 ± 107 pg/mL; HG (20mM): 386 ± 111 pg/mL, *p-value* < 0.05).

Accordingly, these results indicated that although IPCs generated from both N- and DM-Ad-MSCs showed a potential differentiation into IPCs in vitro, N-Ad-MSCs showed greater generation and differentiation into IPCs indicated by morphological changes, gene expression of β-cell markers, and β-cells functional assay while DM-Ad-MSCs showed less potentiality towards the generation of IPCs.

### The effect of N-Ad-MSCs and DM-Ad-MSCs transplantation on hyperglycemia in STZ-induced diabetic rats

#### FBG and BWs of STZ-induced diabetic rats

**Fig. 4:**
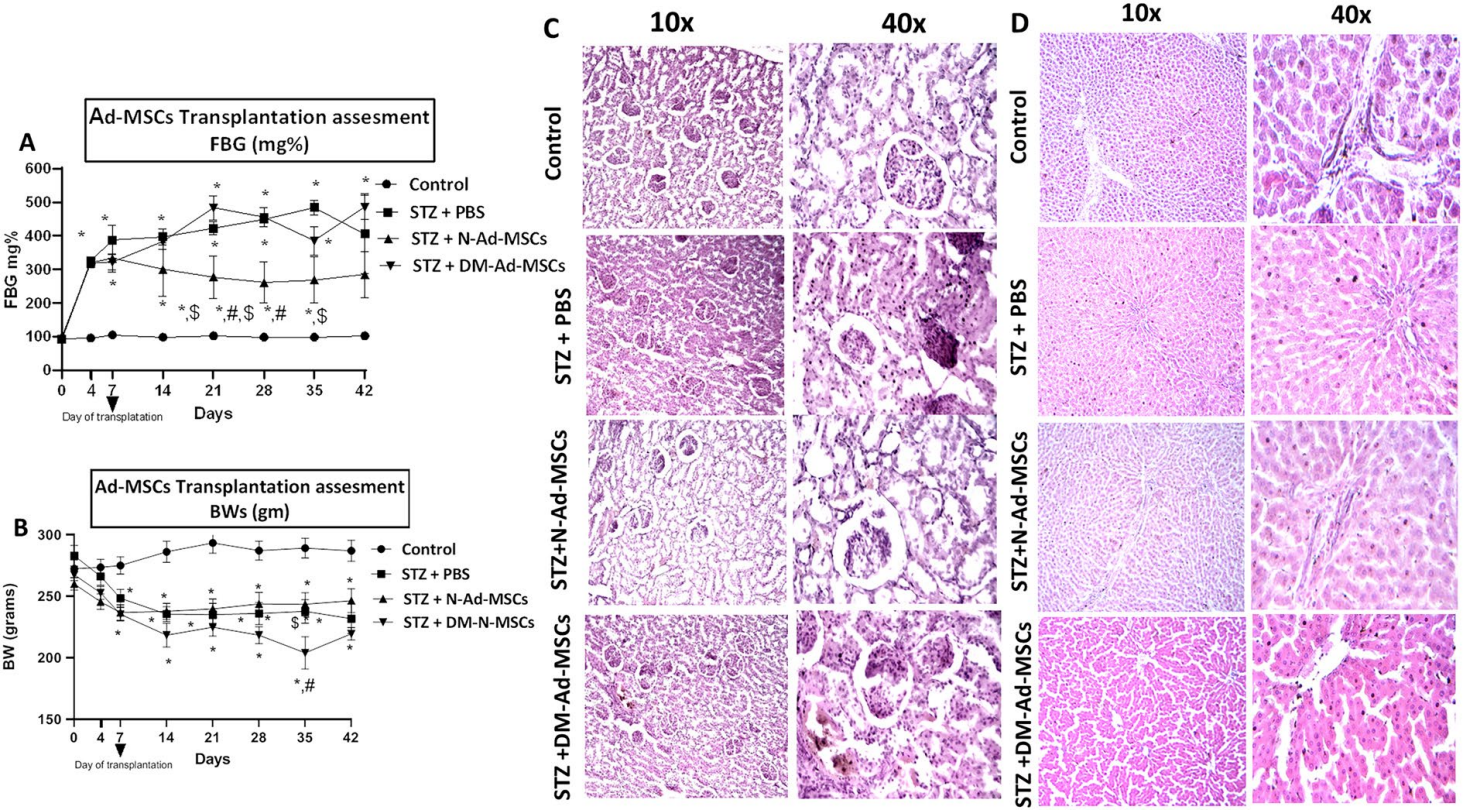
Transplantation of N- and DM- Ad-MSCs in STZ induced diabetic rats: **A** FBG levels of STZ-induced diabetic rats for 42 days post-transplantation of N-Ad-MSCs and DM-Ad-MSCs. **B** Body weights of STZ-induced diabetic rats for 42 days post-transplantation of N-Ad-MSCs and DM-Ad-MSCs. **C** Histopathological examination of pancreas of different study groups (at magnification 10× and 40×). **D** Histopathological examination of liver of different study groups (at magnification 10 × and 40×). Control: non injected group; STZ + PBS, n = 6: group injected with STZ and PBS, n = 6; N-Ad-MSCs: animals injected with STZ and transplanted with N-Ad-MSCs, n = 6; DM-Ad-MSCs: animals injected with STZ and transplanted with DM-Ad-MSCs, n = 6. *: significant different from Control at *p-value* < 0.05. #: significant different from STZ + PBS at *p-value* < 0.05. $: significant different from STZ + DM-Ad-MSCs at *p-value* < 0.05

We employed STZ-induced diabetic rats to investigate the capacity of both N-Ad-MSCs and DM-Ad-MSCs to regulate hyperglycemia in DM. As shown in Fig. 4A, upon the injection of STZ in rats, the FBG began to rise 4 days after the injection, reaching more than (200mg % diabetic). This significant elevation continued till D42 post-injection. Interestingly, transplantation of N-Ad-MSCs caused a significant decline in FBG starting from D21 till D42, however, it failed to reach the control levels. On the other hand, transplantation of DM-Ad-MSCs failed to show this FBG lowering effect. As for BW, injection of STZ caused a significant decline in BWs of the injected animals. On the other hand, transplantation of either N-Ad-MSCs or DM-Ad-MSCs failed to restore this BW loss Fig. 4B. These results indicate that N-Ad-MSCs can be relatively more potent in controlling hyperglycemia in STZ-induced diabetic rats. Means ± SE are provided in Additional file 1: Table S1, S2.

#### Histopathological examination of various organs in transplanted STZ-induced diabetic rats

To further examine the effect of Ad-MSCs transplantation on STZ-induced diabetic rats, we performed a histopathological examination of the pancreata and livers of the injected treated animals. As shown in Fig. 4C, the H&E-stained samples of pancreas taken from normal rats, STZ-induced diabetic rats, and N- or DM-Ad-MSCs treated rats were examined under a light microscope.

The pancreas tissue of the control group (uppermost panel) showed normal islets of Langerhans with pale rounded and ovoid β-cells in the center, embedded in the exocrine portion of the pancreas. The tissue showed marked congestion with an accumulation of fatty vacuoles inside the islets. As for the STZ + PBS group (second panel from top), the pancreas demonstrated a reduction of the islets of Langerhans along with the degeneration and death of component cells whose nuclei appeared densely basophilic and consistently evident karyolysis. Interestingly, the STZ + N-Ad-MSCs group (the third panel from the top) restored the normal islets of Langerhans with its normal pale large round to ovoid shaped containing cells that are embedded in the exocrine portion of the pancreas. In addition, the STZ + DM-Ad-MSCs group (the lowermost panel) showed normal-sized islets of Langerhans but some degeneration of the β cell in the center was noticed.

Liver sections were also H&E stained, as shown in Fig. 4D**,** the control rat section (uppermost panel) displayed a normal histological pattern of hepatic tissue, including normal portal vein, hepatic artery, bile duct, endothelium, hepatic nodules, and sinusoids. In the liver lobules, the hepatocytes are organized in a radial pattern with a gap between each "sinusoid". The hepatic sinusoid has irregular channels and is not coated with intact endothelial cells.

As for the STZ + PBS group, (the second panel from the top) the liver tissues show an increase of vacuolation in the cytoplasm of hepatocytes that appeared as indistinct clear vacuoles that indicate glycogen infiltration in diabetes. In addition, hepatic necrosis and moderate grade of fibrosis are detected especially at the para-biliary ducts. Interestingly, the livers of diabetic rats treated with N-Ad-MSCs, the third panel from the top; showed normal hepatocytes and normal hepatic architecture with mild vacuolation of hepatocytes. The pattern of liver histology showed mild congestion, fatty vacuole, and few islets of lymphocyte infiltration. It also shows a moderate restoration of liver architecture with normal hepatocytes. Mild localized vascular congestion and extravasation were observed with intra-acinar infiltration of lymphocytes. The hepatocytes show mild ballooning degeneration, which is associated with regenerative hepatocytes as indicated by moderately enlarged cells, low nuclear /cytoplasmic ratio, and ballooning. In addition, acidophilic bodies and binucleated hepatocytes are detected. As for the STZ + DM-Ad-MSCs group, the lowermost panel): few focal lymphoplasmacytic infiltrates were shown in periportal and perivascular areas. The hepatocytes show regenerative cellular changes in form of nuclear enlargement, acidophilic bodies, and binucleated hepatocytes. In addition, grade I fibrosis was detected mainly around the biliary ducts and perivascular spaces as well as widening in the intralobular spaces. These results indicate that the transplantation of N-Ad-MSCs could not fully restore the pancreas or liver normal histology which can partially explain the failure of these cells’ transplantation to attain normoglycemia. However, still N-Ad-MSCs can better restore these organs as compared to DM-Ad-MSCs.

#### Immunostaining of insulin in pancreata of various study groups

**Fig. 5:**
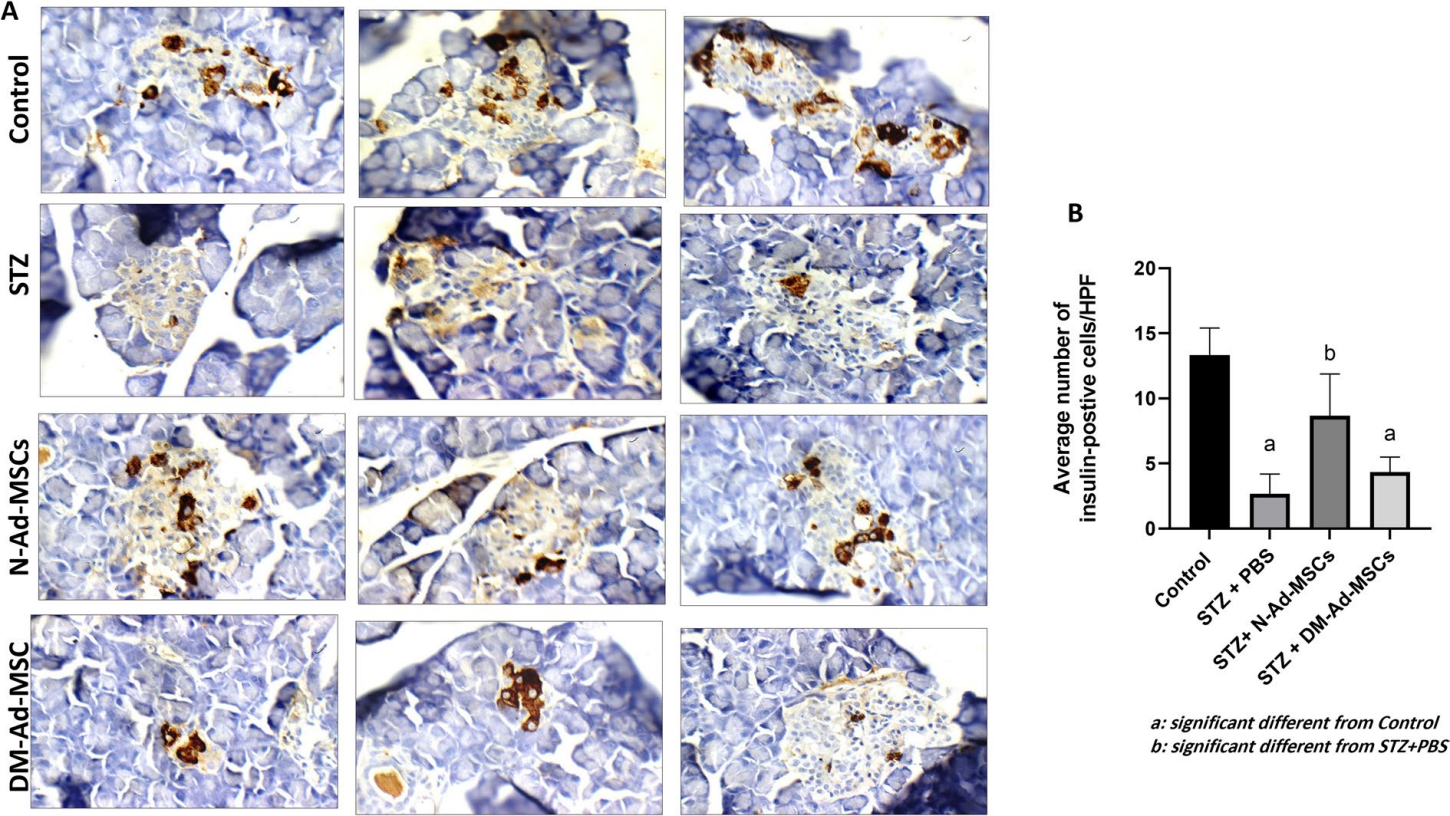
Immunostaining of insulin in pancreata of STZ-induced diabetic rats. **A** Immunostaining of insulin in control non injected group (upper most), STZ-induced diabetic rats (second from top), STZ-induced rats treated with N-Ad-MSCs (third from top) or DM-Ad-MSCs (lowest). **B** Quantification of insulin positive cells/high power field (HPF). Control: non injected group; STZ + PBS, n = 3: group injected with STZ and PBS, n = 3; N-Ad-MSCs: animals injected with STZ and transplanted with N-Ad-MSCs, n = 3; DM-Ad-MSCs: animals injected with STZ and transplanted with DM-Ad-MSCs, n = 3. a: significant different from Control, at *p-value* < 0.05. b: significant different from STZ + PBS, at *p-value* < 0.05

Then, to illustrate the effect of transplantation of N-Ad-MSCs or DM-AdMSCs on insulin expression in pancreata of the transplanted animals, we performed insulin immunostaining on pancreata of all study groups and quantify the number of insulin-positive cells *(ins* +*)* per HPF. As shown in Fig. 5A**,** upon induction of diabetes with STZ, the islets showed decreased insulin staining and less *ins* + cells as compared the control group (control, upper most panel; STZ, second panel from top). Interestingly, transplantation of N-Ad-MSCs restored the insulin staining in the islets and increased *ins* + cells (second panel from bottom). Although transplantation of DM-Ad-MSCs showed increased insulin staining and *ins* + cells, however, this effect failed to reach control levels (lowest most panel). Quantification of the number of *ins* + cells is given in Fig. 5B. These results clearly indicate the better abilities of N-Ad-MSCs to regenerate β-cells in the diabetic rats as compared to DM-Ad-MSCs.

## Discussion

DM is a devastating disease with no radical cure. Lately, MSCs have become the most commonly used cell-based therapy in clinical trials which provide new hope for DM patients either through autologous or allogeneic transplantation [45, 46]***.*** Ad-MSCs gained research interest because they were obtained more conveniently with fewer ethical concerns [3]. Several reports have shown that DM affects the MSCs properties [3, 27]. So, we conducted this study to examine the abilities of Ad-MSCs isolated from STZ induced diabetic rats to generate IPCs in vitro and to alleviate hyperglycemia in vivo in comparison to Ad-MSCs isolated from normal rats (N-Ad-MSCs). Our results showed that N-Ad-MSCs exhibited better potentiality to generate IPCs in vitro either morphologically, genetically or functionally.

Additionally, these N-Ad-MSCs could better alleviate hyperglycemia in diabetic rat, an effect failed to be shown by DM-Ad-MSCs. Furthermore, histopathological examination of pancreas and liver tissues showed that N-Ad-MSCs can restore STZ induced tissue damage as compared to their DM-AD-MSCs counterparts. Moreover, pancreata of diabetic rats transplanted with N-Ad-MSCs showed better restoration of insulin expression as compared to those transplanted with DM-Ad-MSCs. These results clearly demonstrated the devasting effect of DM on the adipose derived MSCs.

Drastic lifestyle changes, insulin injection administration, different classes of drugs, pancreatic transplantation, and immunotherapy are different options in treatment plans for the disease. While these attempts provide some relief to the patient, none of them completely cures DM. Eventually, a new treatment strategy, inspired by MSCs and their unique immunomodulatory and multipotent properties has influenced diabetes therapies that essentially reverse the conditions that cause the disease. Actually, MSCs can alleviate diabetes through several mechanisms including β-cell regeneration for type1 and type2 DM, decreasing insulin resistance in type 2 DM and even decreasing the low-grade inflammatory milieu associated with type2 DM [47]. In this study, we isolated adipose derived MSCs from rat epididymis. Basically, Ad-MSCs are more abundant, with relatively easier accessibility, and easier to isolate than other MSCs derived from different sources such as bone marrow and umbilical cord blood [48]. Interestingly, an in vivo study proved that Ad-MSCs transplantation can restore oxidative stress parameters to control levels, even better than bone marrow derived MSCs [49]. Adipose tissue is now becoming a research focus because it contains adipose-derived stem cells with multipotent ability, in addition to their significant potential for developing regenerative medicine therapies [50]. In our hands, these cells exhibit all MSCs characteristics and excellent culturing properties, an important property for further clinical applications of these cells. These properties were shown to be exhibited also by human Ad-MSCs, a notion which require further investigations [51].

It was reported that DM has deleterious effects on MSCs properties; the main influences of DM are especially hyperglycemia, metabolic disturbance on Ad-MSCs, ROS alteration and immunosuppression [3, 27]. In our study, N-Ad-MSCs clearly showed better differentiation to IPCs in vitro and better control of hyperglycemia in vivo*.* Interestingly, Ad-MSCs can restore insulin expression in pancreata of the transplanted animals. This clearly indicate the abilities of these MSCs to regenerate β-cells either directly or through secreting various signaling molecules that help in restoring the pancreatic β-cells [52, 53]*.* Consequently, N-Ad-MSCs showed better restoration of insulin than DM-MSCs. These results came in accordance with another study that compared the proliferation activity, viability, morphology, mitochondrial dynamics, mRNA and miRNA expression, and secretory activity of adipose-derived MSCs derived from non-diabetic and diabetic donors. They noticed that Ad-MSCs from diabetic patients had reduced stemness with decreased viability and proliferative potential in addition to an impairment in anti-oxidants protection [54]. Furthermore, another study showed that obese, metabolic syndrome and DM patients' MSCs had increased apoptosis and limited multipotency of MSCs [55, 56]. These facts must be considered before MSCs can be used in endocrinology practice especially in autologous transplantation of MSCs in diabetic patients.

One clinical implication of our study is the consideration of the autologous transplantation of Ad-MSCs in diabetes. Clinical case studies using autologous treatment of adipose-derived MSCs are still very limited and challenging because of the deleterious effect of DM on ad-MSCs as mentioned above [3]. Interestingly, a clinical case study suggests autologous Ad-MSCs translated to insulin-secreting islet-like cell aggregates **(**ICAs) as an effective alternative strategy for the treatment of juvenile diabetes where a 21-year-old female with type 1 diabetes had received treatment with ICAs derived from autologous Ad-MSCs and the patient’s insulin requirement was significantly reduced with no side effects were observed [57].

In another clinical study [58], 7 patients were enrolled (2 with T1DM and 5 with T2DM) using autologous Ad-MSCs and ICAs to manage high blood glucose levels and it had been ended with blood glucose levels were minimally reduced in T1DM patients. They suggested three possible explanations which are that T1DM patients are very lean and do not have much adipose, only a small amount of lipoaspirate is collected from them, and the implanted ICAs may not survive for long due to autoimmune attack, so to protect implanted ICAs, they recommended allogenic ICAs derived from MSCs and a biocompatible cell impermeable scaffold but in the case of type 2 diabetes, the results are more impressive due to increased insulin secretion from ICAs. These studies, among others, indicate that consideration of autologous transplantation of Ad-MSCs and the exact mechanisms of actions including the immunomodulatory effects of MSCs on type 1 and type 2 DM still require further investigations especially in clinical settings.

Although MSCs are considered the most promising source of allogenic cell therapy for DM, yet, there isn't enough evidence to definitively prove that MSCs can develop into mature, functioning cells or islet-like organoids [59]***.*** This was clearly demonstrated by the inability of these cells to restore the normal blood glucose levels or insulin expression in the islets of the transplanted animals. This can be considered as a limitation for this study. Recent research studies examined the hypothesis of the possibility of MSCs differentiation into IPCs [59, 60]. This was based in part on the observation that insulin and other pancreatic transcription factors are upregulated in differentiating MSCs. However, the presence of such markers as Ngn-3, and NKX6.1 are not proof of fully matured cells, as some of these factors are found to be expressed also during MSC in vitro expansion [61] another limitation to be considered in further investigations. The findings of this study, aligned with the previously mentioned observations, showed that N-Ad-MSCs failed to restore the fasting blood glucose level of the treated group back to the control level. It is noteworthy here to consider assessment of serum insulin, as shown by Yousef and colleagues, 2022 [62] together with blood glucose and histopathological alterations as a marker to assess the functional efficiency of transplanted MSCs in treatment of DM either in preclinical or clinical studies. Therefore, generation of mature IPCs from MSCs still require further investigations including genetic modifications or use of new differentiating factors such as obestatin to better generate IPCs [63].

## Conclusion

In summary, this study proved that adipose tissue is an accessible source for MSCs isolation. Ad-MSCs showed their differentiation potential into IPCs. Ad-MSCs isolated from diabetic rats exhibit less ability to generate IPCs in vitro and alleviate hyperglycemia in vivo as compared to their normal Ad-MSCs counterpart. This can be attributed to the deleterious effect of DM on the MSCs. This may hinder the autologous use of MSCs in the treatment of diabetic patients and encourage more research on MSCs sources that are more suitable for allogeneic transplantation**.**

### Supplementary Information

**Additional file 1: Table S1.** Fasting Blood Glucose (FBG) of the study groups. **Table S2.** Body weights (BW) of the study groups.

## Data Availability

All the data generated is included in the manuscript and any further data is available upon request from the corresponding author.

## Abbreviations

β-ME: β-Mercaptoethanol
ANOVA: One-way analysis of variance
DM: Diabetes mellitus
DM-Ad-MSCs: Diabetic-Ad–MSCs
DMSO: Dimethyl sulfoxide
DMEM/F12: Dulbecco's modified eagle medium/nutrient mixture F-​12
DTZ: Dithizone
EDTA: Ethylenediaminetetraacetic acid
ELISA: Enzyme-linked immunosorbent assay
FACS: Fluorescence-activated cell sorting
FBG: Fasting blood glucose
FBS: Fetal bovine serum
FITC: Fluorescein-isothiocyanate
GSIS: Glucose-stimulated insulin secretion test
H&E: Hematoxylin and eosin
HG: High glucose
IL: Interleukin
Ins-1: Insulin-1
IPCs: Insulin-producing cells
ISCT: International society for Cell and gene therapy
KRB: Kreb’s Ringer bicarbonate
LG: Low glucose
MafA: Musculoaponeurotic fibrosarcoma oncogene homolog A
MSCs: Mesenchymal stem cells
N-Ad-MSCs: Normal-Ad–MSCs
NA: Nicotinamide
NEUROD1: Neurogenic differentiation 1
Ngn-3: Neurogenin-3
NKX2-2: NK2 related transcription factor related, locus 2
NK: Natural killer
Nkx6.1: Homeobox protein Nkx6
OD: Optical density
PBS: Phosphate Buffered Saline
PE: Phycoerythrin
PFA: Paraformaldehyde
ROS: Reactive oxygen species
RT: Room temperature
SE: Standard error of the mean
STZ: Streptozotocin
SVF: Stromal vascular fraction
T1DM: Type 1 diabetes mellitus
T2DM: Type 2 diabetes mellitus
TNF-α: Tumor necrosis factor-alpha
