# Supplementary material for: The effect of diabetes mellitus on differentiation of mesenchymal stem cells into insulin-producing cells
Source: Biol Res. 2024 May 2;57:20. doi: 10.1186/s40659-024-00502-4 (PMC11067316; doi:10.1186/s40659-024-00502-4)
Supplement: Supplementary file 1 — Additional file 1: Table S1. Fasting Blood Glucose (FBG) of the study groups. Table S2. Body weights (BW) of the study groups. [file 40659_2024_502_MOESM1_ESM.docx]

**Table S1: Fasting Blood Glucose (FBG) of the study groups**

| **STZ+DM-Ad-MSCs** | **STZ+N-Ad-MSCs** | **STZ +PBS** | **Control** | **Days** |
| --- | --- | --- | --- | --- |
| 92.4 ±1.2 | 100.4 ±2.6 | 92.6 ±1.4 | 93.40 ±2.54 | **D0** |
| 318.3 ±13.5 | 319.6 ±15.1 | 324.4 ±12.5 | 95.80 ±4.37 | **D4** |
| 321.6 ±24.3 | 333.0 ±41.9 | 387.4 ±43.7 | 105.40 ±2.60 | **D7** |
| 383.4 ±24.6 | 299.6 ±80,3 | 397.2 ±23.9 | 98.00 ±4.07 | **D14** |
| 483.2 ±36.7 | 277.2 ±62.9 | 422.8 ±18.8 | 102.40 ±3.59 | **D21** |
| 454.8 ±28.7 | 261.4 ±61.1 | 449.4 ±11.4 | 98.80 ±5.28 | **D28** |
| 385.2 ±42.3 | 268.6 ±69.5 | 484.0 ±22 | 98.60 ±7.59 | **D35** |
| 485 ±35.9 | 284.8 ±68.5 | 406.2 ±118 | 102.20 ±1.46 | **D42** |

All data are expressed as means ± standard error of means, n=6

**Table S2: Body weights (BW) of the study groups**

| **STZ+DM-Ad-MSCs** | **STZ+N-Ad-MSCs** | **STZ +PBS** | **Control** | **Days** |
| --- | --- | --- | --- | --- |
| 267.60 ±4.6 | 260.0 ±5.1 | 283.0 ±8.4 | 272.4 ±7.2 | **D0** |
| 252.80 ±5.2 | 245.5 ±6.3 | 266.2 ±7.3 | 273.6 ±6.7 | **D4** |
| 235.40 ±5.5 | 236.8 ±6.3 | 248.4 ±7.2 | 275.0 ±7.1 | **D7** |
| 218.40 ±9.6 | 237.8 ±6.5 | 235.6 ±6.1 | 286.2 ±8.6 | **D14** |
| 224.90 ±7.2 | 239.8 ±8.0 | 235.2 ±9.2 | 293.4 ±8.4 | **D21** |
| 218.48 ±6.96 | 243.8 ±9.4 | 236.0 ±8.9 | 287.2 ±7.7 | **D28** |
| 204.00 ±13.19 | 243.6 ±9.4 | 237.8 ±9.6 | 289.2 ±8.0 | **D35** |
| 219.60 ±5.07 | 246.4 ±9.7 | 231.8 ±12.0 | 287.0 ±8.7 | **D42** |

All data are expressed as means ± standard error of means, n=6
